# Circulating exosomal microRNA-203 is associated with metastasis possibly via inducing tumor-associated macrophages in colorectal cancer

**DOI:** 10.18632/oncotarget.20009

**Published:** 2017-08-07

**Authors:** Yuki Takano, Takaaki Masuda, Hisae Iinuma, Rui Yamaguchi, Kuniaki Sato, Taro Tobo, Hidenari Hirata, Yosuke Kuroda, Sho Nambara, Naoki Hayashi, Tomohiro Iguchi, Shuhei Ito, Hidetoshi Eguchi, Takahiro Ochiya, Katsuhiko Yanaga, Satoru Miyano, Koshi Mimori

**Affiliations:** ^1^ Department of Surgery, Kyushu University Beppu Hospital, Beppu, Japan; ^2^ Department of Surgery, Jikei University School of Medicine, Tokyo, Japan; ^3^ Department of Surgery, Teikyo University, Tokyo, Japan; ^4^ Human Genome Center, Institute of Medical Science, University of Tokyo, Tokyo, Japan; ^5^ Department of Pathology, Kyushu University Beppu Hospital, Beppu, Japan; ^6^ Division of Molecular and Cellular Medicine, National Cancer Center Research Institute, Tokyo, Japan

**Keywords:** miR-203, exosome, tumor-host interaction, tumor-associated macrophage, colorectal cancer

## Abstract

A primary tumor can create a premetastatic niche in distant organs to facilitate the development of metastasis. The mechanism by which tumor cells communicate with host cells to develop premetastatic niches is unclear. We focused on the role of microRNA (miR) signaling in promoting metastasis. Here, we identified *miR-203* as a signaling molecule between tumors and monocytes in metastatic colorectal cancer (CRC) patients. Notably, high expression of serum exosomal *miR-203*, a major form in circulation, was associated with distant metastasis and an independent poor prognostic factor, whereas low expression in tumor tissues was a poor prognostic factor in CRC patients. We also found that exosomes carrying *miR-203* from CRC cells were incorporated into monocytes and *miR-203* could promote the expression of M2 markers *in vitro*, suggesting *miR-203* promoted the differentiation of monocytes to M2-tumor-associated macrophages (TAMs). In a xenograft mouse model, *miR-203*-transfected CRC cells developed more liver metastasis compared to control cells. In conclusion, serum exosomal *miR-203* expression is a novel biomarker for predicting metastasis, possibly via promoting the differentiation of monocytes to M2-TAMs in CRC. Furthermore, we propose the concept of site-dependent functions for *miR-203* in tumor progression.

## INTRODUCTION

Distant metastasis is the leading cause of mortality in cancer patients, including those with CRC which is one of the most common malignant tumors worldwide [1] and has a poor prognosis despite recent advances in diagnosis and treatment [2]. Metastasis is a multistep process that requires tumor cells to leave the primary tumor through intravasation, survival in the circulation and extravasation into distant organs that provide an appropriate microenvironment.

The ‘seed and soil hypothesis’ of malignancy proposed that the distribution of metastasis from cancers is not based on chance alone, and that the microenvironment of the potential metastatic ‘soil’ could either promote or prevent the primary ‘seed’ from growing [3, 4]. This “seed and soil” hypothesis is fundamental to the concept of the premetastatic niche whereby tumors prepare defined organs for metastasized cells [5]. It is now well recognized that the formation of a premetastatic niche is crucial for the development of metastasis.

We recently reported that increased production of C-C motif chemokine 2 (*CCL2*) due to downregulation of F-box and WD repeat domain containing 7 (*FBXW7*) in bone marrow (BM)-derived stromal cells promoted the formation of premetastatic niches through recruitment of myelo-monocytic cell-derived suppressor cells and TAMs, thereby promoting metastatic tumor growth [6]. Our finding provided strong evidence that host cells play a critical role in tumor metastasis through the formation of premetastatic niches. However, the mechanisms by which tumor cells communicate with the host cells to develop a premetastatic niche are poorly understood. Understanding this mechanism should open up a new field of cancer therapy through the targeting of tumor-host interaction.

We have focused on circulating miRs, a class of small noncoding RNAs that are mainly carried by exosomes in circulation [7]. They may act as critical intercellular signals between cancer cells and host BM cells, promoting the development of premetastatic niches. Substantial evidence suggests that miRs play an important role in multiple aspects of tumor progression [8] and can work as messengers in intracellular communication through extracellular vesicles such as exosomes [9]. miRs regulate one-third of all human protein-coding genes by binding to the 3’-untranslated region of a target mRNA [10]. miRs affect a wide variety of biological processes, including proliferation, differentiation, cell fate determination, apoptosis, and cancer by mediating mRNA destabilization or translational repression. We previously determined the clinical significance of miR expression in peripheral blood and BM, and identified a correlation between miRNA expression and distant metastasis [11-14]. These studies provided clues as to the mechanism of communication between tumors and host cells, including TAMs in CRC.

In this study, we identified *miR-203* as a signaling molecule between tumor and host immune cells. Moreover, we assessed the clinical significance of *miR-203* expression in both exosomes in serum and tumor tissues in CRC patients. Next, we examined if *miR-203* from tumor cells could promote distant metastasis by affecting host immune cells *in vitro* and *in vivo*. Finally, we propose that *miR-203* may have dual functions in CRC progression.

## RESULTS

### An association between disseminated tumor cells (DTCs) and monocytes was shown by genes downstream from *miR-203* in CRC patients with distant metastasis

**Figure 1:**
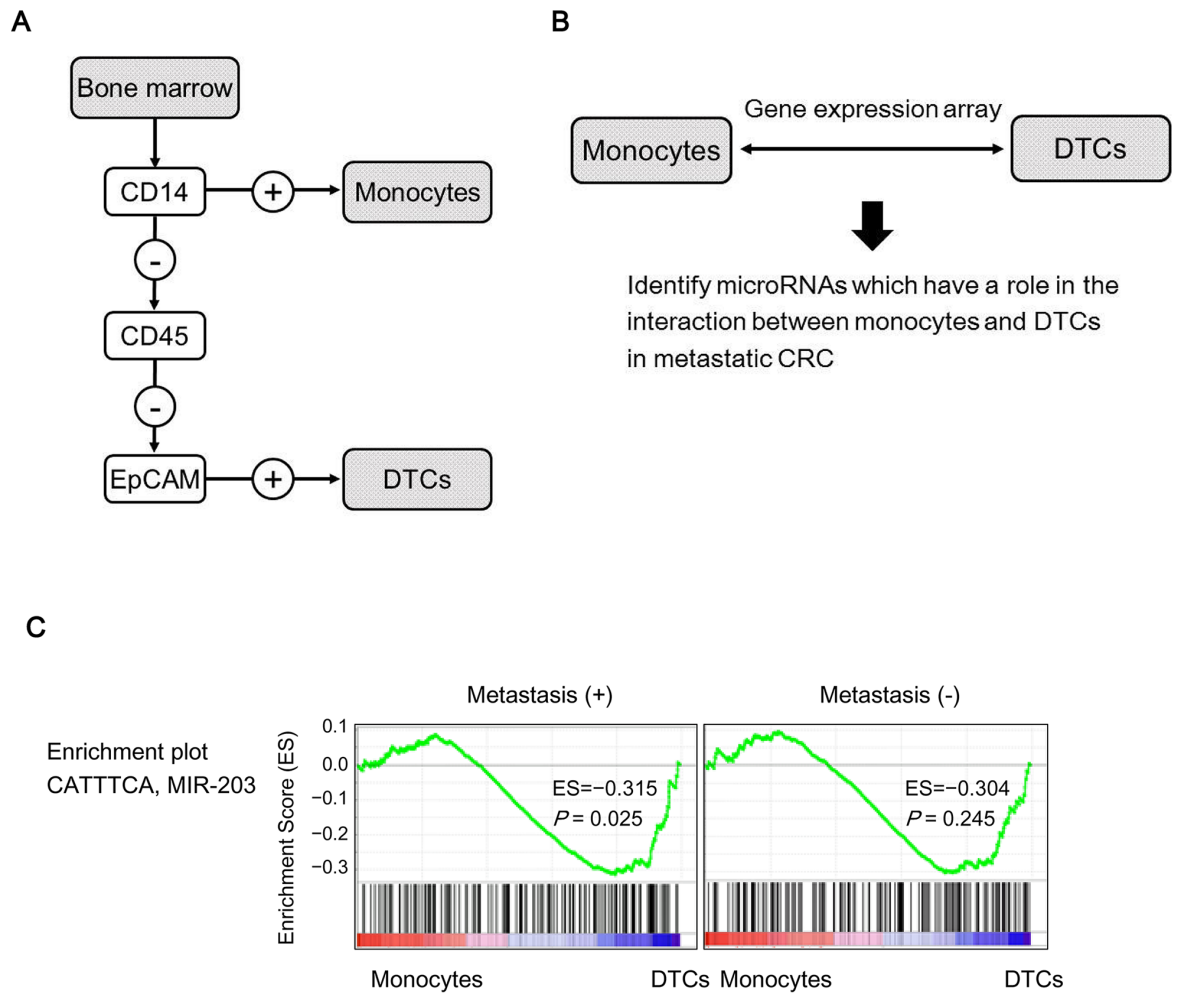
Identification of miR-203 as a potential intercellular signal between DTCs and monocytes **(A)** Isolation of DTCs and monocytes from BM using MACS. **(B)** Gene expression array analysis identified genes that showed conflicting expression in DTCs and monocytes in metastatic CRC patients. **(C)** GSEA with DTCs and monocytes from CRC patients with or without metastasis was performed to identify upstream miRs for the genes that were identified by gene expression analysis (B).

We used a MACS system to separate *CD14*^+^ cells (monocytes) and *CD14*^−^/*CD45*^−^/*EpCAM*^+^ cells (DTCs) from BM of CRC patients with or without distant metastasis (Figure 1A). After extracting mRNAs, gene expression microarray analysis was performed on the monocytes and DTCs for each sample (Figure 1B).

For screening miRs that may have a significant role in the interaction between monocytes and DTCs in metastatic CRCs, we compared expression levels of mRNAs between monocytes and DTCs either within the group of CRC patients with metastasis (4 cases: metastasis group) or within the group of those without metastasis (6 cases: non-metastasis group) as follows. We searched miR-target gene sets in each of which the member genes were significantly enriched in genes whose expression levels were statistically different between in DTCs and monocytes within the metastasis group but not within the non-metastasis group. For that purpose, we analyzed mRNA expressions by gene set enrichment analysis (GSEA) software as described in MATERIALS AND METHODS.

As the result of the screening, we found *miR-203* as a candidate; the *miR-203* target gene set was negatively enriched in DTCs relative to monocytes (*P*=0.025) within the metastasis group but not significant within the non-metastasis group (*P*=0.245) (Figure 1C). These data imply that *miR-203* from DTCs could affect monocytes in BM during the process of metastasis.

### Clinical significance of exosomal *miR-203* expression in serum in CRC

Based on GSEA data, we hypothesized that *miR-203* produced by cancer cells was associated with metastatic events through its regulatory linkage with host cells. In other words, we proposed that circulating *miR-203* affected the formation of premetastatic niches, which is considered essential for tumor metastasis. To pursue this possibility, we examined the clinical significance of exosomal *miR-203* in serum because it is a major fraction of miR in circulation and exosomes are considered an intercellular messenger [7, 15].

**Figure 2:**
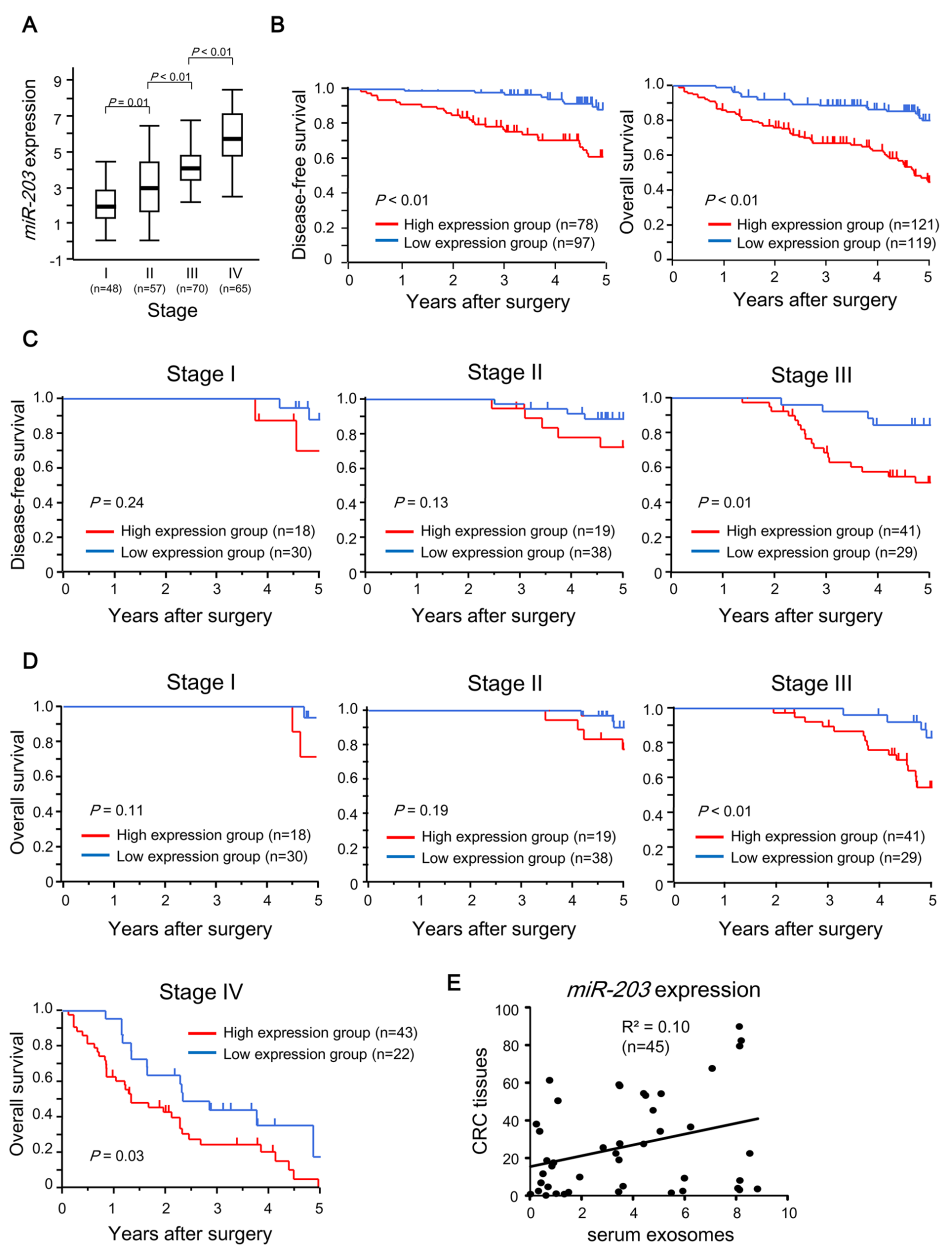
Prognostic significance of exosomal miR-203 expression in serum in CRC patients **(A)** Exosomal *miR-203* expression in serum in different TNM stages of CRC. **(B)** Kaplan-Meier survival curves of CRC patients according to *miR-203* expression level. Left: DFS of patients who underwent curative surgery. Right: OS of all patients. **(C)** Subgroup analyses for DFS of patients with Stages I, II or III who underwent curative surgery according to tumor stage. **(D)** Subgroup analyses for OS of all patients according to tumor stage. **(E)** Correlation of *miR-203* expression between in serum exosome and in CRC tissues.

First, the expression of serum exosomal *miR-203* was analyzed in 240 CRC patients. The values ranged from 0.01 to 8.48 (median, 3.89). The median expression levels of *miR-203* in each TNM stage were 1.96, 2.94, 4.09 and 5.71, respectively (Figure 2A). The level of *miR-203* expression was significantly upregulated in a TNM stage-dependent manner (Figure 2A). Next, we assessed the prognostic significance of serum exosomal *miR-203* expression using all 240 cases for overall survival (OS) and 170 cases with Stages I, II and III with curative surgery for disease-free survival (DFS). Cases were divided into two groups as described in Materials and Methods. The cut-off value was 3.89. High exosomal *miR-203* expression was significantly associated with poor DFS and OS (*P* < 0.01, Figure 2B). Then, univariate and multivariate analyses for prognosis were performed (Table 1). Clinicopathological factors that were found to be prognostic factors in univariate analysis were included in the multivariate analysis. Notably, multivariate analysis demonstrated that exosomal *miR-203* expression in serum was an independent prognostic factor for both DFS and OS in CRC patients. Moreover, we performed subgroup analyses for prognosis according to tumor stage. As shown in Figure 2C and 2D, the trends indicated that high expression group exhibited poorer prognosis. Statistically significant differences in DFS or OS were noted among only patients with an advanced stage: DFS, stage III (*P* = 0.01) or OS, stages III (*P* < 0.01) and IV (*P* = 0.03), respectively. In Figure 2E, no statistical correlation of *miR-203* expression was shown between in serum exosomes and tumor tissues (R^2^ = 0.10).

**Table 1:** Univariate and multivariate analysis of prognostic factors for OS and DFS of CRC patients

Variables	Univariate analysis		Multivariate analysis	
	HR (95% CI^a^)	*P*	HR (95% CI)	*P*
**OS** **(n=240)**				
Histology (Not well/Well)	1.29 (0.81-2.02)	0.28		
Tumor size (≧5cm/<5cm)	1.59 (1.01-2.50)	0.04	1.22 (0.76-1.97)	0.42
Depth of invasion (≧SS^c^/≦MP^b^)	2.58 (1.47-4.91)	<0.01	1.10 (0.54-2.37)	0.79
Venous invasion (+/-)	3.12 (1.82-5.71)	<0.01	1.71 (1.88-3.46)	0.11
Lymphatic invasion (+/-)	2.60 (1.66-4.11)	<0.01	1.60 (0.95-2.70)	0.08
Lymph node metastasis (+/-)	2.06 (1.31-3.27)	<0.01	1.02 (0.61-1.71)	0.94
Liver matastasis (+/-)	11.54 (7.07-18.89)	<0.01	5.59 (3.04-10.53)	<0.01
Peritoneal dissemination (+/-)	3.11 (1.37-6.18)	<0.01	2.79 (1.16-5.97)	0.02
Serum CEA (≧5.0ng/ml/<5.0ng/ml)	4.56 (2.78-7.79)	<0.01	1.97 (1.07-3.70)	0.03
Serum CA19-9 (≧37U/ml/<37U/ml)	3.41 (2.16-5.37)	<0.01	0.99 (0.58-1.71)	0.98
Exosomal *miR-203* in serum (high/low)	3.53 (2.15-6.02)	<0.01	2.27 (1.31-4.09)	<0.01
**DFS** **(n=170)**				
Histology (Not well/Well)	1.13 (0.56-2.22)	0.73		
Tumor size (≧5cm/<5cm)	0.81 (0.39-1.62)	0.56		
Depth of invasion (≧SS/≦MP)	1.47 (0.72-3.23)	0.30		
Venous invasion (+/-)	2.98 (1.41-7.07)	<0.01	2.65 (1.24-6.33)	0.01
Lymphatic invasion (+/-)	1.74 (0.85-3.44)	0.13		
Lymph node metastasis (+/-)	2.72 (1.38-5.59)	<0.01	1.67 (0.83-3.50)	0.15
Serum CEA (≧5.0ng/ml/<5.0ng/ml)	2.27 (1.15-4.48)	0.02	2.14 (1.07-4.31)	0.03
Serum CA19-9 (≧37U/ml/<37U/ml)	0.89 (0.30-2.11)	0.81		
Exosomal *miR-203* in serum (high/low)	4.24 (2.05-9.62)	<0.01	3.56 (1.70-8.16)	<0.01

^a^CI, confidence interval; ^b^MP, muscularis propria; ^c^SS, subserosa.

Next, the relationship between clinicopathological factors and exosomal *miR-203* expression in serum was examined using the 240 patients. Compared with the low *miR-203* expression group, the high expression group showed a higher incidence of venous invasion (*P* = 0.02), lymph node metastasis (*P* < 0.01), distant metastasis (*P* < 0.01) including liver metastasis (*P* < 0.01), lung metastasis (*P* < 0.05) and peritoneal dissemination (*P* = 0.05) and higher TNM stage (*P* < 0.01) (Table 2). Tumor cells may acquire the ability to secret exosomal *miR-203* in serum during tumor progression.

**Table 2:** Relationship between clinicopathological factors and exosomal miR-203 expression in serum in CRC

Variables	Low (n=119) number (%)	High (n=121) number (%)	*P*
Sex			0.81
Female	47 (39.5)	46 (38.0)	
Male	72 (60.5)	75 (62.0)	
Tumor size (cm)			0.59
<5	70 (58.8)	67 (55.4)	
≧5	49 (41.2)	54 (44.6)	
Histology			0.81
Well	71 (59.7)	74 (61.2)	
Not Well	48 (40.3)	47 (38.8)	
Depth of invasion			0.11
≦MP^a^	46 (38.7)	34 (28.1)	
≧SS^b^	73 (61.3)	87 (71.9)	
Venous invasion			0.02
(-)	54 (45.4)	37 (30.6)	
(+)	65 (54.6)	84 (69.4)	
Lymphatic invasion			0.06
(-)	82 (68.9)	69 (57.0)	
(+)	37 (31.1)	52 (43.0)	
Lymph node metastasis			<0.01
(-)	77 (64.7)	56 (46.3)	
(+)	42 (35.3)	65 (53.7)	
Distant metastasis			<0.01
(-)	97 (81.5)	78 (64.5)	
(+)	22 (18.5)	43 (35.5)	
Liver metastasis			<0.01
(-)	115 (96.6)	79 (65.3)	
(+)	4 (3.4)	42 (34.7)	
Lung metastasis			0.04
(-)	114 (95.8)	107 (88.4)	
(+)	5 (4.2)	14 (11.6)	
Peritoneal dissemination			0.05
(-)	108 (90.8)	117 (96.7)	
(+)	11 (9.2)	4 (3.3)	
Others			0.10
(-)	117 (98.3)	113 (93.4)	
(+)	2 (1.7)	8 (6.6)	
TNM Stage			<0.01
I, II	68 (57.1)	37 (30.6)	
III, IV	51 (42.9)	84 (69.4)	

Correlation was analyzed by Fisher’s exact test.

^a^MP, muscularis propria; ^b^SS, subserosa.

These clinical data indicated that high expression of exosomal *miR-203* in serum was strongly associated with metastasis followed by poor prognosis, findings that would support our hypothesis that *miR-203* is a link between cancer and host cells in metastatic progression. However, the signaling role played by serum exosomal *miR-203* is not well understood.

### *miR-203* released from CRC cells *in vitro* stimulated differentiation of monocytes to M2 macrophages

**Figure 3:**
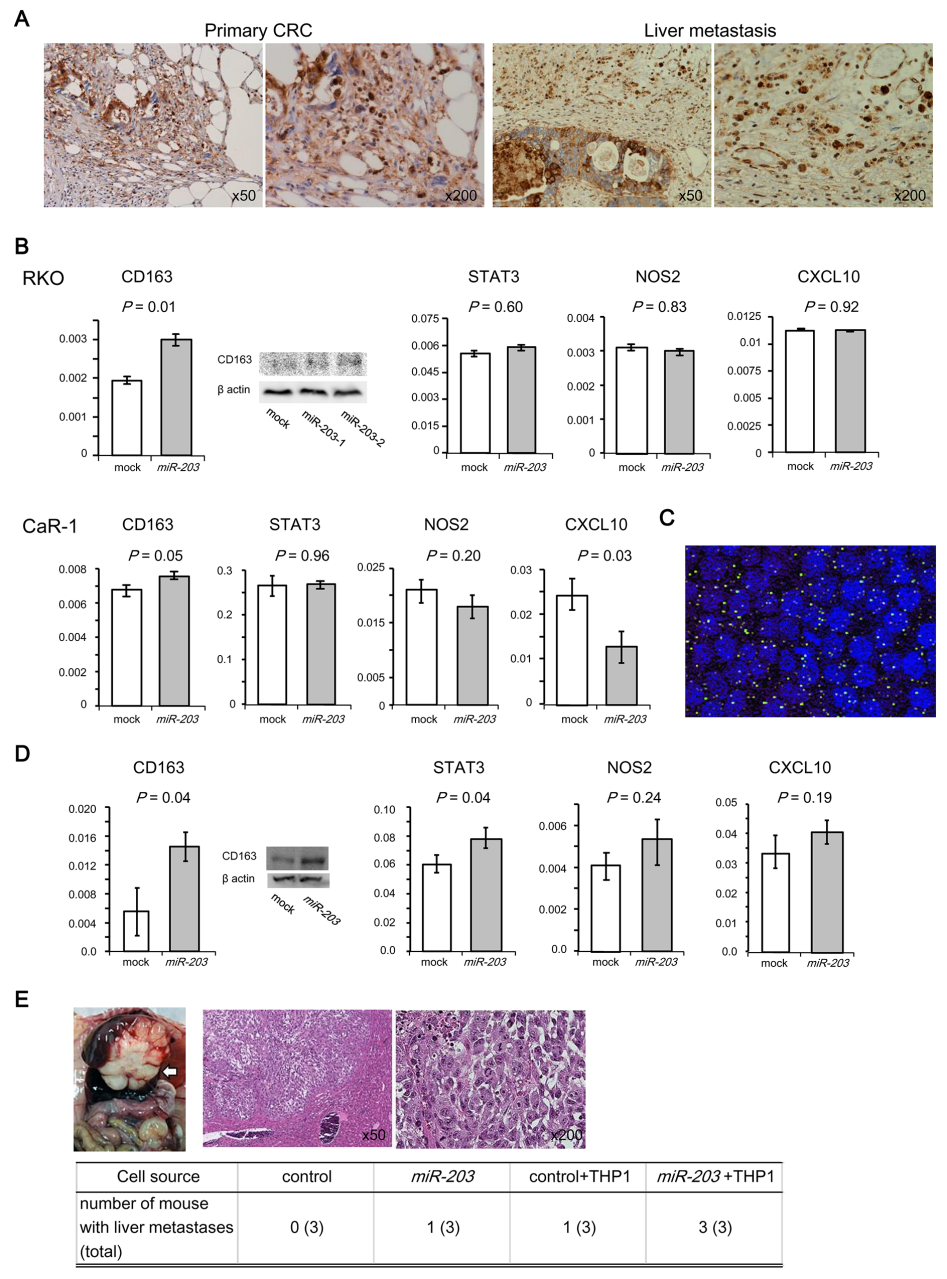
Effect of miR-203 on the differentiation of monocytes to M2 macrophages and the metastatic potential of CRC cells **(A)** Immunostaining for arginase 1 (M2 macrophage marker) in primary or liver metastatic lesion of CRC. Most of the macrophages and some of the cancer cells were positively stained. **(B)** Expression of M2 (*CD163*, *STAT3*) and M1 (*NOS2*, *CXCL10*) markers of THP-1s that were co-cultured with CRC cell lines (RKO and CaR-1). RT-qPCR, Western blot. **(C)** Labeling of exosomes with PKH67. Exosomes (green) were incorporated into THP-1s (blue). **(D)** Expression of M2 and M1 markers by THP-1 cells that were transfected with *miR-203*. RT-qPCR, Western blot. **(E)** Xenograft liver metastatic mouse model. Animals were injected with *miR-203*-transfected RKO cells or control RKO cells through the splenic vein with or without THP-1 cells. A representative image of the metastatic liver (upper left panel), histologic examination of liver metastases with H&E staining (upper right panels) and the number of mice with liver metastasis (bottom panel).

TAMs are a critical component of the tumor microenvironment, and they affect tumor growth, immune suppression, and metastasis [16, 17]. In particular, M2 macrophages, a subtype of TAMs, are thought to promote tumor development. Thus, *miR-203* released from cancer cells may facilitate distant metastasis through directed differentiation of monocytes to M2 macrophages that constitute the premetastatic niche. In fact, immunohistochemical analysis revealed that many M2 macrophages (arginase 1-positive macrophages) infiltrated not only primary tumor tissues but also metastatic liver lesions in CRC (Figure 3A).

To pursue this finding, we conducted RT-qPCR experiments to assess if *miR-203* affected the differentiation of the monocytic cell line THP-1. Thus, we followed the expression of M1 markers (*NOS2* and *CXCL10*) and M2 markers (*CD163* and *STAT3*). First, co-culture experiments using *miR-203*-transfected CRC cell lines (RKO and CaR-1) and THP-1 were performed in transwell culture plates allowing free exchange of culture medium. *miR-203* was overexpressed in the *miR-203*-transfected CRC cells (Supplementary Figure 1A). The expression of *CD163* in THP-1 co-cultured with *miR-203*-transfected RKO or CaR-1 was higher than with control cells (Figure 3B). Moreover, CXCL10 was downregulated in THP-1 co-cultured with *miR-203*-transfected CaR-1 (Figure 3B).

Next, exosomes from the supernatant of *miR-203*-transfected cells were added to the culture medium of THP-1. The expression of exosomal *miR-203* in the supernatant of *miR-203*-transfected cells was higher than from that of the control cells (Supplementary Figure 1A). Exosomes were incorporated into 27 of 46 THP-1 cells (Figure 3C). Finally, THP-1 was transfected with a *miR-203* expression vector or empty vector. *miR-203* was overexpressed in *miR-203*-transfected THP-1 (Supplementary Figure 1B). The expression of the M2 marker in *miR-203*-transfected THP-1 was higher than the control (Figure 3D). These observations suggest that *miR-203* released from CRC cells could direct the differentiation of monocytes into M2 macrophages.

### *miR-203* from circulating CRC cells promoted liver metastasis in a xenograft model

To evaluate the metastatic potential of *miR-203*-transfected CRC cells, we conducted a liver metastasis assay using xenograft mice in which the cells were injected with or without THP-1 cells into the splenic vein (Figure 3E). Control cells alone did not produce liver nodules, whereas *miR-203*-transfected RKO cells generated nodules in 1 of 3 mice and *miR-203*-transfected RKO with THP-1 developed nodules in all 3 mice. The appearance of the livers of representative animals is shown in Figure 3E. Histological analysis showed that all liver nodules contained cancer cells, indicating there was a liver metastasis. These data indicate that *miR-203* from circulating CRC cells promoted liver metastasis.

### Overexpression of *miR-203* did not affect proliferation, invasive or migration capacity of cancer cells *in vitro*

To examine the proliferation, invasive or migration potential of *miR-203*-transfected CRC cells, which are associated with metastatic potential, MTT proliferation assays and *in vitro* invasion or migration assays were performed using *miR-203*-transfected RKO and CaR-1 cells. There was no significant difference in either assay in comparisons of *miR-203*-transfected cells and the control cells (Supplementary Figure 2A, 2B, 2C). This observation supports our hypothesis that *miR-203* promotes metastasis by affecting host cells as described above.

### Clinical significance of *miR-203* expression in tumor tissues from CRC

Our study demonstrated that exosomal *miR-203* in serum could function as an oncogene in CRC by altering host cells and thereby promoting metastasis. However, there are several lines of evidence that *miR-203* can also work as a tumor suppressor in CRC. To determine the function of *miR-203* in tumor tissues, we examined the clinical significance of *miR-203* expression in CRC tissues using 2 independent datasets.

**Figure 4:**
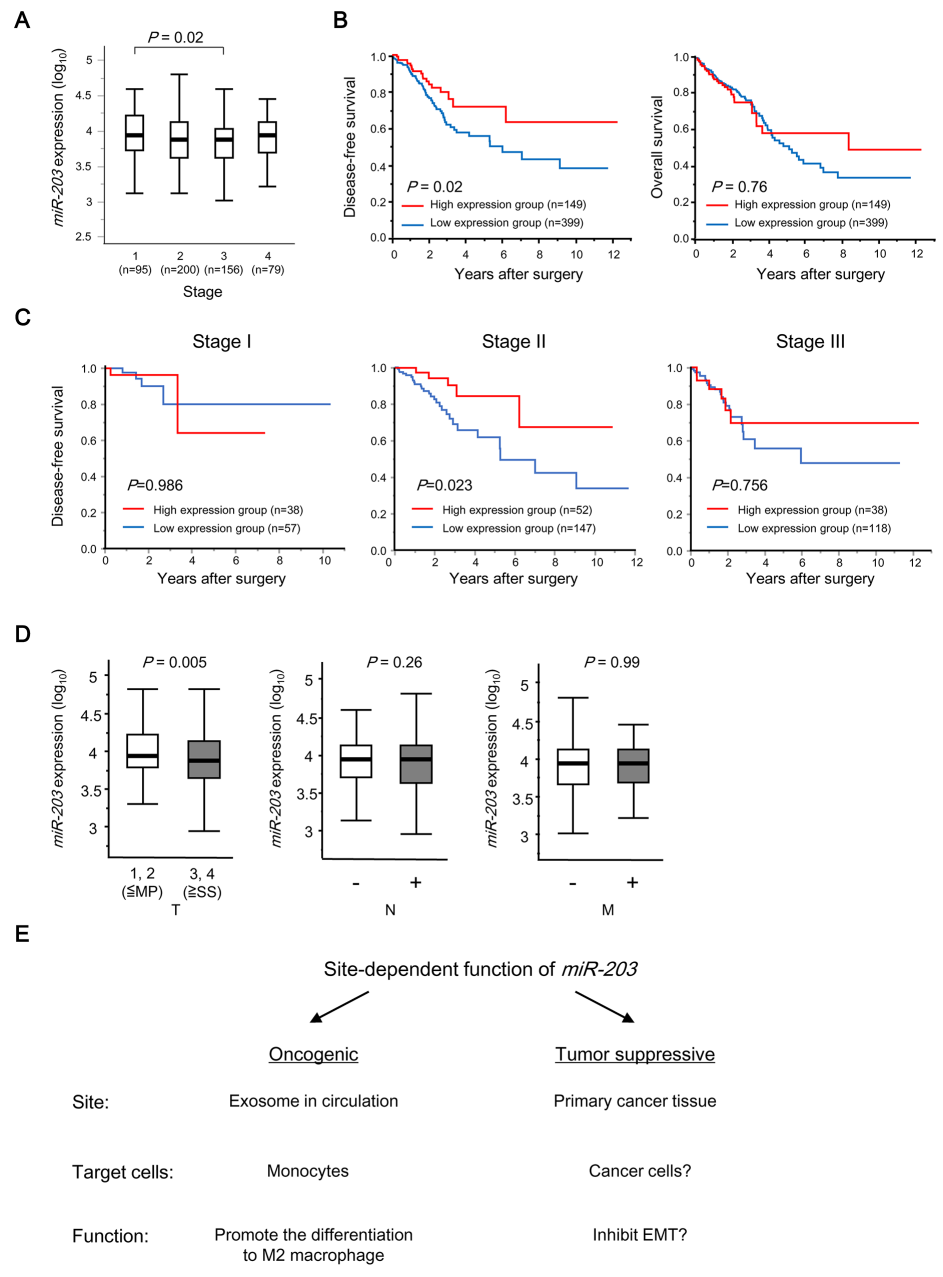
Clinical significance of miR-203 expression in tumor tissues in CRC patients **(A)** *miR-203* expression in different TNM stages of CRC from TCGA datasets. **(B)** Kaplan-Meier OS curves of CRC patients based on *miR-203* expression in TCGA datasets. Left: DFS; Right: OS. **(C)** Subgroup analyses of DFS of the patients who underwent curative surgery according to tumor stage. **(D)** The relationship between *miR-203* expression and TNM factors in CRC patients in TCGA datasets. **(E)** Schematic depiction of site-dependent functions of *miR-203* in CRC progression.

First, we investigated the clinical significance of *miR-203* expression in tumor tissues using the The Cancer Genome Atlas (TCGA) database. The values ranged from 2.57 to 5.04 (median, 3.88). The cut-off value of *miR-203* expression was set at 4.12 as described in MATERIALS AND METHODS. The median expression levels of *miR-203* in the TNM stages were 3.94, 3.88, 3.88 and 3.94, respectively (Figure 4A). Interestingly, the low *miR-203* expression group showed poorer DFS than the high group (*P* = 0.02, Figure 4B). Subgroup analyses for DFS by tumor stage are shown in Figure 4C. This trend was exhibited in all Stages except Stage I, but the statistical difference in DFS was noted among only patients with Stage II (*P* = 0.023). Furthermore, *miR-203* expression was negatively correlated with a greater depth of invasion (T) (*P* = 0.005) (Figure 4D).

Next, we examined the clinical significance of pre-*miR-203* expression in tumor tissues using Kyushu datasets. Pre-*miR-203* was used in place of mature *miR-203* for RT-qPCR because only CRC tissue cDNA was available for the expression analysis in our institute and an analysis using CRC cell lines revealed that there was a high correlation of expression between mature *miR-203* and pre-*miR-203* (R^2^ = 0.69, Supplementary Figure 3A, 3B). The values of pre-*miR-203* expression in tumor tissues ranged from 0.094 to 18.8 (median, 0.791). The cut-off value of *miR-203* expression was set at 1.89 as described in MATERIALS AND METHODS. Supplementary Figure 3C showed that pre-*miR-203* expression in tumor tissues was significantly higher than in normal tissues (*P* =0.01), suggesting reactive expression of pre-*miR-203* to suppress tumor development. Furthermore, similar results with that from the TCGA dataset were obtained, which showed that pre-*miR-203* expression did not change with TNM stage (Supplementary Figure 3D) and the low pre-*miR-203* expression group had poorer DFS than the high group (*P* = 0.03, Supplementary Figure 3E). Multivariate analysis demonstrated that low expression of pre-*miR-203* was an independent prognostic factor for DFS in CRC patients (Supplementary Table 1). Furthermore, pre-*miR-203* expression was negatively correlated with a greater depth of invasion (*P* =0.05, Supplementary Table 2).

Collectively, these 2 independent clinical datasets supported the observation that *miR-203* in tumor tissues may function as a tumor suppressor for CRC progression as reported elsewhere.

## DISCUSSION

In this study, we identified *miR-203* as a link between tumor cells and monocytes through a bioinformatic analysis, and demonstrated that high expression of exosomal *miR-203*, a major form in circulation, was associated with high stage of tumor pathological aggressiveness and progression, including lymph node metastasis, venous invasion, distant metastasis and a higher TNM stage in CRC patients. Importantly, high exosomal *miR-203* expression in serum was an independent poor prognostic factor, consistent with a recent similar study of circulating *miR-203* in CRC [18]. Moreover, we found that intravenous transfer of CRC cells that overexpressed *miR-203* had a higher potential for liver metastasis in a xenograft model. Plus, *miR-203* promoted the differentiation of monocytes to M2 macrophages *in vitro*. These observations suggest that circulating tumor-derived exosomal *miR-203* could facilitate distant metastasis by inducing host M2-TAMs to develop a premetastatic niche. Thus, exosomal *miR-203* in serum may facilitate CRC progression by acting as an intercellular messenger between tumor and host cells. A further study using a genetically-engineered mouse model will be needed to validate this.

Recently, it was reported that tumor-derived exosomes containing miRs could initiate the formation of premetastatic niches by inducing host cells to promote metastasis in pancreatic and breast cancer [19-21]. Furthermore, an experimental mouse model showed that integrins in tumor-derived exosomes determined organotrophic metastasis [22]. Here, we have provided clinical evidence to support these observations, suggesting exosomes or their contents could be used for early diagnosis of metastasis and for targeted therapy in cancer treatment.

Interestingly, there is evidence that *miR-203* in tumor tissues acts as a tumor suppressor by promoting the mesenchymal to epithelial transition [23, 24] and inhibiting proliferation or invasion through direct targeting of certain oncogenes [25] [26-31], although the prognostic role in cancers remains elusive [32]. In fact, 2 independent datasets showed that the expression of *miR-203* in tumor tissues was inversely correlated with the depth of invasion and the high expression group had a better prognosis compared to the low expression group. Moreover, lower pre-*miR-203* expression in tumor tissues was an independent poor prognostic factor in Kyushu dataset. Hence, our results demonstrate that *miR-203* in tumor tissues works as a tumor suppressor whereas exosomal *miR-203* in serum acts as an oncogene by affecting host cells to promote metastasis. *MiR-155* has also been shown experimentally to have conflicting functions in mouse breast cancer [33]. Recently, studies have focused on the contrasting roles of miRs in oncogenesis and tumor suppression [32, 34].

Here, we propose that tumor-derived miR has site-dependent functions that promote tumor development (Figure 4E). miRs may produce an overall net oncogenic or tumor suppressive effect [34]. Thus, miR localization should be considered for clinical cancer screening or anti-cancer therapy for targeting miR. Further examination of the functions of miR in various sites in cancer patients will be required to better understand its apparently conflicting functions in cancer progression.

The mechanism by which *miR-203* promotes the differentiation of monocytes to M2 macrophages is unclear. Suppressor of cytokine signaling 3 *(SOCS3)*, a target of *miR-203* [35], reportedly promotes activation of M1 macrophages and reduced expression of *SOCS3* in macrophages resulted in the induction of M2 macrophages [36-39]. Therefore, tumor-derived exosomal *miR-203* may reduce expression of *SOCS3* followed by induction of M2 macrophages and inhibition of M1 macrophages. Further work is needed to clarify this.

In summary, we demonstrated that *miR-203* constitutes a link between tumor and host cells and, furthermore, exosomal *miR-203* in serum could be a novel biomarker for predicting metastasis possibly via its promotion of monocyte differentiation to M2-TAMs and subsequent formation of premetastatic niches. Finally, we provide important clinical evidence that *miR-203* has dual functions in CRC progression.

Preventing metastasis by eliminating the formation of premetastatic niches through inhibition of intercellular signal miRs could be a new attractive approach for cancer treatment.

## MATERIALS AND METHODS

### Clinical samples

All clinical samples were obtained from CRC patients treated at Teikyo University Hospital or Kyushu University Beppu Hospital with written informed consent. This study was approved by each institutional review board, and the Ethics and Indications Committee. Clinicopathological factors and clinical stage were classified using the TNM system of classification. All data for the samples, including gender, tumor size and depth of invasion, lymphatic invasion, lymph node metastasis, vascular invasion, liver metastasis, peritoneal dissemination, distant metastasis, clinical stage, histological grade and serum tumor markers (CEA and CA19-9) were obtained from the clinical records. We treated patients in accordance with the Japanese Society of Cancer of the Colon and Rectum Guidelines for the Treatment of Colorectal Cancer. OS was defined as the time between the date of diagnosis and the date of death. DFS was defined as the length of time after surgical treatment for cancer during which the patient survived without a sign of recurrence.

We used a total of 240 serum samples from CRC patients treated at Teikyo University Hospital between 2005 and 2012 for extraction of exosomes. Forty-five corresponding matched CRC tissues were available. The median follow-up period was 54 months (range, 3–67 months). Patients treated with radiotherapy or chemotherapy prior to surgery were not included in the study. Blood samples were collected from patients before surgery.

Tumor tissues and paired normal tissues were obtained from 88 CRC patients. Those patients underwent primary tumor resection at Kyushu University Beppu Hospital and affiliated hospitals between 1992 and 2007. The average overall survival time after resection was 4.03 years in all patients. Tumor recurrence was observed in 24 of 88 patients.

Ten BM samples were obtained from CRC patients who underwent primary or metastatic tumor resection at Kyushu University Beppu Hospital in 2011. Among the 10 samples, 4 were from patients with liver metastases, and 6 were from those without metastases (Supplementary Table 3).

### Gene expression microarray analysis

Cellular RNA expression levels were profiled using an Agilent DNA microarray system as described previously [40]. In brief, cyanine (Cy)-labeled cDNA was prepared using T7 linear amplification, after which it was fragmented and hybridized to an oligonucleotide microarray (Whole Human Genome 4 × 44 Agilent G4112F). Fluorescence intensities were obtained using an Agilent DNA microarray scanner and processed by quantile normalization [41]. The microarray data will be uploaded in Gene Expression Omnibus datasets (National Center for Biotechnology Information, Bethesda, MD, USA).

### Analysis of TCGA

We obtained *miR-203* expression in tumor tissues, TNM classification and survival data from 548 CRC cases in TCGA from the Broad Institute’s Firehose (http://gdac.broadinstitute.org/runs/stddata__2016_01_28/data/COADREAD/20160128/). The miR sequencing data were normalized with quantile normalization [41].

### GSEA

GSEA analysis with miR target gene sets, which were defined in the collection of motif gene sets (C3) in the Molecular Signatures Database (MSigDB), was performed for gene expression data set from the metastatic CRC patient group and that from the non-metastatic CRC patient group, separately [42-44]. To make a pre-ranked gene list for each group as an input list for GSEA software, we examined gene expression differences between DTCs (*CD14*-/*CD45*-/*EpCAM*+ cells) and monocytes (*CD14*+ cells) with t-test for each gene within a group. We used the value -I_i_ log_10_ P_i_ as the metric for the *i*-th gene in each pre-ranked gene list, where *P*_*i*_ is the p-value of the t-test and *I*_*i*_ is the indictor variable for which *I*_*i*_ =1 when the t-statistic > 0 and *I*_*i*_ = −1 otherwise.

### Cell culture

Ten different human CRC cell lines, CaR-1, RKO, Colo205, Colo320DM, DLD1, HCT116, Lovo, SW480, SW620 and HT29 and the human monocyte line THP-1 were obtained from the Cell Resource Center for Biomedical Research Institute of Development, Aging and Cancer, Tohoku University. They were maintained in RPMI 1640 supplemented with 10% FBS at 37°C in a 5% humidified CO_2_ atmosphere.

To assess if the production of cellular factors such as exosomes from *miR-203*-transfected cells affected the differentiation of THP-1, co-culture experiments were carried out in a transwell culture plate (Corning-Costar, Acton, MA, USA) allowing free exchange of culture medium and solutes. *miR-203*-transfected cells or the control and THP-1 were seeded in the lower and upper wells, respectively.

### Isolation of BM fractions

BM was aspirated and separated into 3 fractions (*CD14*+, *CD14*-/*CD45*+, and *CD14*-/*CD45*-/*EpCAM*+) as previously described [14]. *CD14*, *CD45* and *EpCAM* are monocyte, hematopoietic, and epithelial marker, respectively. In brief, BM aspirates were obtained from the sternum using a BM aspiration needle under general anesthesia before surgery. BM cells were separated into 3 fractions using a three-step automagnetic-activated cell separation system (MACS) by MACS Cell Separators (Miltenyi Biotec, Bergisch Gladbach, Germany). *CD14*+, *CD14*-/*CD45*+ and *CD14*-/*CD45*-/*EpCAM*+ cell fractions were collected using *CD14*, *CD45*, and *EpCAM* microbeads according to the manufacturer’s instructions (Miltenyi Biotec), yielding monocytes, lymphocytes, and DTCs, respectively. Each fraction was mixed with Isogen-LS (Nippon Gene, Toyama, Japan) and stored at -80°C.

### Isolation of exosomes

Exosomes were isolated from serum or supernatant as described previously [13]. In brief, peripheral blood and BM were collected and centrifuged at 3000 r.p.m. for 10 min at 4°C to collect serum. Supernatants of cultured cells were collected after incubation with exosome-depleted FBS for 3 days. The supernatant was collected and centrifuged at 2000 g for 10 min at room temperature, and at 12 000 g for 30 min followed by filtration through a 0.22-mm filter to remove cell debris. Sera or supernatants (1.0 mL) intended for RT–qPCR were ultracentrifuged at 100 000 g for 70 min at 4°C. The pellets containing exosome were washed with PBS. The putative exosome fraction was measured for its protein content using a Quant-iTTM Protein Assay with a Qubit® 2.0 Fluorometer (Invitrogen). Nanoparticle tracking analysis was performed using the NanoSight LM10HS with a blue laser system (NanoSight, Amesbury, UK) on isolated exosomes diluted 500-fold with PBS for analysis.

### Extraction of total RNA

Serum exosomes were extracted for total RNA using the miRNeasy serum/plasma kit (Qiagen, Venlo, Netherlands) according to the manufacturer’s protocol. The RNA quality was assessed using an Agilent 2100 Bioanalyzer (Agilent Technologies, Santa Clara, Calif). Frozen tissue specimens or subconfluent cultured cell lines were homogenized, and total RNA was extracted using the modified acid-guanidine-phenol-chloroform method with Isogen or Isogen-LS according to the manufacturer’s instructions.

### Reverse transcription-quantitative PCR (RT-qPCR)

RT-qPCR assessments of *miR-203*, *miR-16*, and *RNU6B* were performed as described previously [12]. In brief, *miR-203*, *miR-16* and *RNU6B*-specific cDNAs were synthesized using gene-specific primer sets according to the TaqMan® Micro-RNA Reverse Transcription Kit protocol (Applied Biosystems, Foster City, Calif., USA). qPCR was performed using PCR LightCycler® 480 Probes Master (Applied Biosystems). *miR-203*, *miR-16* (as an internal control for vesicular *miR-203* [12]), and *RNU6B* (as an internal control for intracellular *miR-203*) were purchased from Applied Biosystems. Relative quantification of miR expression was calculated using the 2-ΔΔCt method [45]. The raw data were presented as the relative quantity of target microRNA, normalized with respect to *miR-16* or *RNU6B* and relative to a calibrator sample.

RT-qPCR assessments of pre-*miR-203*, *CD163*, *STAT3*, *NOS2*, *CXCL10*, and *GAPDH* were performed as previously described [46]. In brief, RT was performed with random hexamers using M-MLV reverse transcriptase (Invitrogen, Carlsbad, CA). qPCR was performed with LightCycler® FastStart DNA Master SYBR Green I (Roche Diagnostics). The raw data were presented as the relative quantity of target genes, normalized with respect to *GAPDH*. The primer sequences for RT-PCR were as follows: pre-*miR-203*, forward 5’- TGGTCCTAAACATTTCACAA -3′ and reverse 5’- TCCAGTGGTTCTTAACAGTTC -3’; *CD163*, forward 5’- CCATGGGAGCGAAGAATC-3′ and reverse 5’- CTCCACGCACTCTTATTCTATCTTC-3’; *STAT3* forward 5’- CCCTTGGATTGAGAGTCAAGA-3′ and reverse 5’- AAGCGGCTATACTGCTGGTC-3’; *NOS2*, forward 5’- TTCCTTACGAGGCGAAGAAG-3′ and reverse 5’- TCAGAGCGCTGACATCTCC-3’; *CXCL10*, forward 5’- CCCCACGTTTTCTGAGACAT-3′ and reverse 5’- TGGCAGTTTGATTCATGGTG-3′ and *GAPDH*, forward, 5’-TTGGTATCGTGGAAGGACTC-3′ and reverse, 5’-AGTAGAGGCAGGGATGATGT-3’.

### Immunohistochemical staining

Immunohistochemical studies of arginase 1 (M2 marker) were performed on specimens available from 10 CRC cases from Kyushu University using the avidin-biotin-peroxidase method (LSAB2 kit; Dako, Kyoto, Japan) on formalin-fixed, paraffin embedded tissues. All sections were counterstained with hematoxylin. The primary antibody against arginase 1 was used at dilutions of 1:1000. Rabbit polyclonal antibody to arginase 1 was purchased from Bioss Antibodies (Woburn, MA). Histological analysis was independently performed by an experienced research pathologist at Kyushu University.

### Western blot analysis

Total protein was extracted from samples with RIPA buffer. Western blotting was performed as described previously [42]. Rabbit polyclonal antibody to *CD163* and mouse monoclonal antibodies to *β-actin* were purchased from Santa Cruz Biotechnology (Santa Cruz, CA). The dilutions were 1:500. The blots were developed with horseradish peroxidase-linked anti-rabbit or anti-mouse immunoglobulin (Promega, Inc., Madison, WI). Signals were detected using SuperSignal (Pierce, Inc., Rockford, IL).

### Labeling exosomes with PKH67

Exosomes derived from RKO were isolated as described above and labeled using PKH67 Fluorescent Cell Linker kits (Sigma-Aldrich, St. Louis, MO) according to the manufacturer’s instructions and described elsewhere [47]. To examine the uptake of exosomes into THP-1, the cells were plated in 8-well chamber slides (1 x 10^4^ cells/well) using each medium. After 24 h, the slides were washed three times in PBS, and each medium containing PKH67-labeled exosomes or a negative control sample was added to each well. Cells were cultured for 48 h at 37°C in an atmosphere of 5% CO_2_. After incubation, the slides were washed three times with PBS, and 4% paraformaldehyde solution was then added to the slides. These were fixed for 10 min at room temperature. The slides were washed three times in PBS again. Nuclear staining was performed using a ProLong Gold antifade reagent with DAPI (Life Technologies) and the slide was covered with cover glass. Finally, the cells were visualized under a confocal laser scanning microscope LSM710 (Carl Zeiss, Oberkochen, Germany) under the same conditions. The green dots on THP-1 (blue cells) were regarded as exosomes incorporated into THP-1.

### Transfection assays and establishment of CRC cell lines stably transfected with pre-miR-203

The backbone plasmid pcDNA6.2-GW/EmGFP-miR was from the Block-iT Pol II miR RNAi Expression Vector Kit (Invitrogen). The plasmids pcDNA6.2-GW/EmGFP-pre-*miR-203* (pCMV-pre-*miR-203*) containing pre-*miR-203* and pcDNA6.2-GW/EmGFP-miR-neg (pCMV-N) containing an unrelated insert were constructed as described in the manual for the Block-iT Pol II miR RNAi Expression Vector Kit. The sequence of mature *miR-203* was GUGAAAUGUUUAGGACCACUAG (hsa-*miR-203*a-3p).

pCMV-pre-*miR-203* and pCMV-mir-neg were transfected into the cell lines using Lipofectamine 3000 (Life Technologies, Inc., Tokyo, Japan) according to the manufacturer’s instructions. Then, stably-transfected cells expressing mature *miR-203* (*miR-203*-transfected cells) were selected with G418 (800 μg/mL) treatment followed by sorting for GFP by MACS. A pCMV-mir-neg -transfected clone of each cell line was used for the control.

### MTT assay

Cell proliferation was evaluated by performing MTT assays using a Cell Proliferation Kit 1 (Roche Applied Science, Penzberg, Germany) according to the manufacturer’s instructions as described previously [40]. In brief, miR203-transfected cells and mock-transfected cells were seeded at 3,000 cells per well in triplicate 96-well trays in 100 μL medium. The color reaction was quantitated using an automatic plate reader, Immuno-Mini NJ-2300 (Nihon InterMed, Tokyo, Japan), at 570 nm with a reference filter of 650 nm. Each independent experiment was carried out three times.

### *In vitro* invasion or migration assay

Cell invasive or migration capacities were assessed using the BD BioCoat Tumor Invasion System, 24 Multiwell or the BD Falcon FluoroBlok 24 Multiwell Insert System (BD Bioscience) according to the manufacturer’s instructions as described previously [40]. In brief, *miR-203*-transfected cells and mock-transfected cells (1.0 x 10^5^ cells/well) were placed in the upper chamber, and the lower chamber was filled with 750 μL of RPMI 1640 with 10% FBS as a chemoattractant, and incubated in a humidified atmosphere (37°C and 5% CO_2_). After a 48 h incubation, the upper chamber was transferred into a second 24-well plate containing 500 μL of 4 μg/mL calcein AM in HBSS in each well, and the plates were incubated for an additional 1 h (37°C and 5% CO_2_). Invasive cells that migrated through the membrane were evaluated in a fluorescence plate reader at excitation/emission wavelengths of 485/535 nm. Invasiveness was measured as the percentage of fluorescence of an invasive fibrosarcoma cell line (HT-1080) that served as a control. Each independent experiment was performed three times.

### Liver metastasis studies in a xenograft mouse model

To analyze the role of *miR-203* in tumor metastasis, 6- to 8-week-old BALB/c nu/nu female mice were injected intrasplenically with 1 × 10^6^ *miR-203*-transfected or the control RKO cells suspended in 100 μL PBS. Three weeks later, the mice were euthanized and the livers were analyzed for metastatic lesions by counting or measuring liver weights. The number of liver nodules that were larger than 5 mm in diameter was also determined as metastases. All of the animal studies were approved by the ethics committee of Kyushu University and all animal procedures were performed in compliance with the Guidelines for the Care and Use of Experimental Animals established by the Committee for Animal Experimentation of Kyushu University; these guidelines conform to the ethical standards required by Japanese law and also comply with the guidelines for the use of experimental animals in Japan.

### Statistical analysis

For clinical analysis, cases were divided into two groups using the minimum P value approach based on the *miR-203* expression level, which is a comprehensive method to identify the optimal risk separation cutoff point in continuous gene expression measurements for survival analysis in multiple datasets [48]. Associations between the variables were tested with the Mann–Whitney U test or Fisher’s exact test. The degree of linearity was estimated by Pearson’s correlation co-efficient. Survival curves were drawn according to the Kaplan-Meier method and survival analysis was carried out by the log-rank test when two curves were being compared. Cox proportional hazards regression was used to determine univariate and multivariate hazard ratios for OS and DFS. A two-sided *P*≦0.05 was deemed statistically significant. Statistical analyses were performed using JMP Pro 12 software (SAS Institute, Cary, N.C., USA).

## SUPPLEMENTARY MATERIALS FIGURES AND TABLES




