# Supplementary material for: Circulating exosomal microRNA-203 is associated with metastasis possibly via inducing tumor-associated macrophages in colorectal cancer
Source: Oncotarget. 2017 Aug 7;8(45):78598–613. doi: 10.18632/oncotarget.20009 (PMC5667985; doi:10.18632/oncotarget.20009)
Supplement: Supplementary file 1 [file oncotarget-08-78598-s001.pdf]

Circulating exosomal microRNA-203 is associated with metastasis possibly via inducing tumor-associated macrophages in colorectal cancer

SUPPLEMENTARY MATERIALS

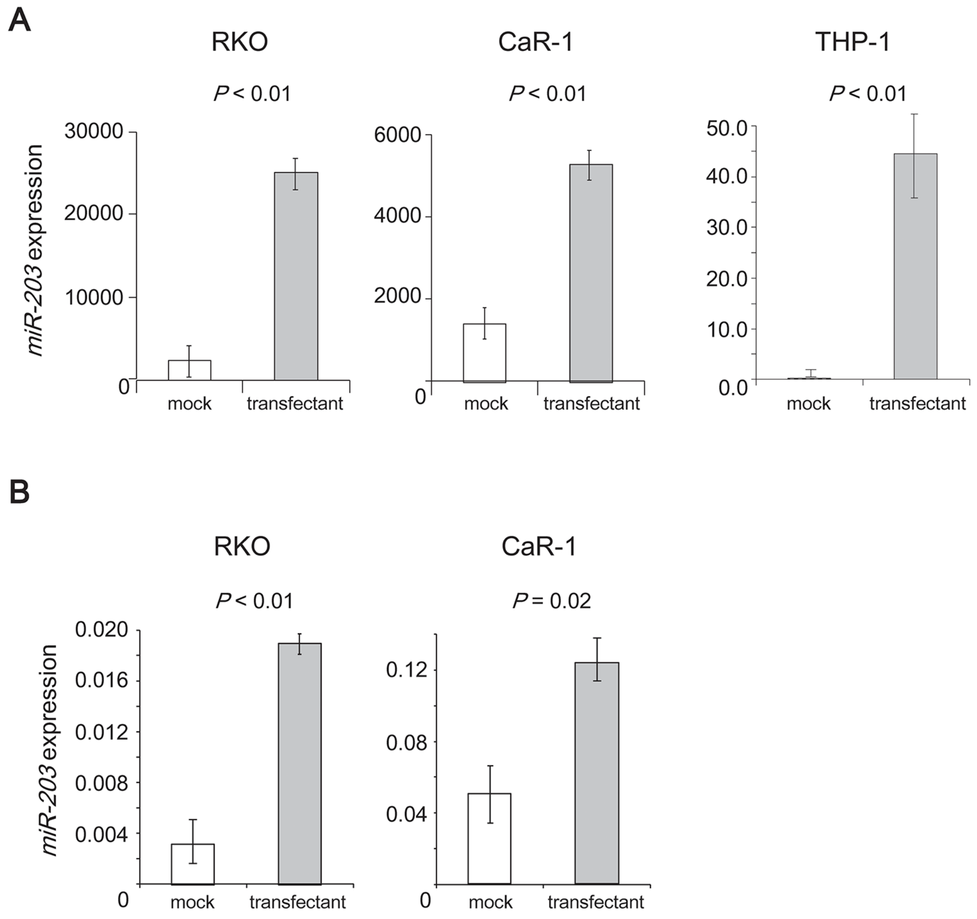

Supplementary Figure 1: Pre-*miR-203*-transfected CRC cell lines and THP-1. **(A)** *miR-203* expression in the culture medium of pre-*miR-203*-transfected RKO and CaR-1 by RT- qPCR. **(B)** Confirmation of *miR-203* upregulation in pre-*miR-203*-transfected RKO and CaR-1, and THP-1 by RT-qPCR.

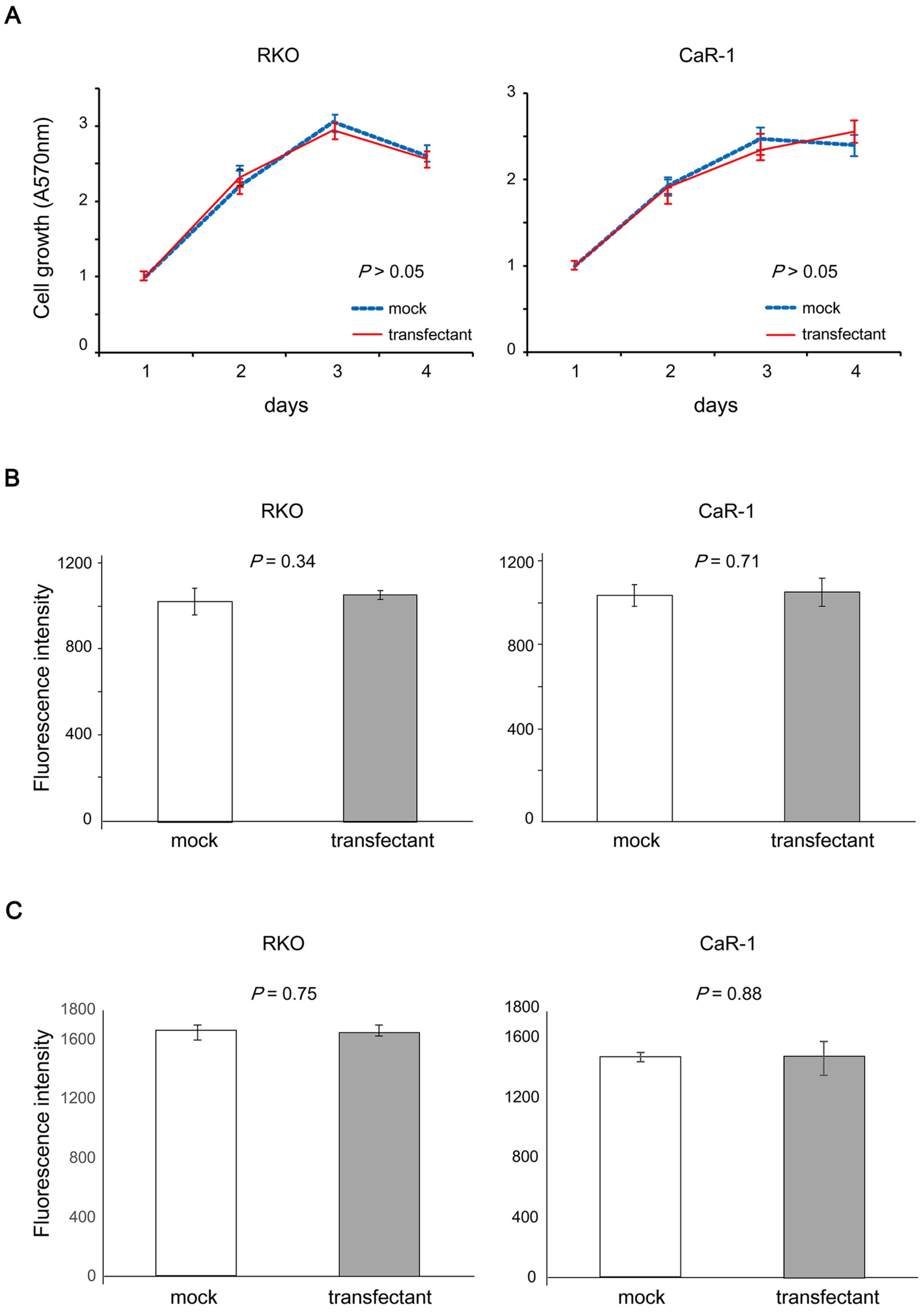

Supplementary Figure 2: Overexpression of *miR-203* did not affect proliferation, invasive or migration capacity of CRC cell lines *in vitro*. (A) MTT assay. **(B)** *In vitro* invasion assay, transfectant: miR-203-transfected CRC cells; mock: the control CRC cells. **(C)** *In vitro* migration assay, transfectant: miR-203-transfected CRC cells; mock: the control CRC cells.

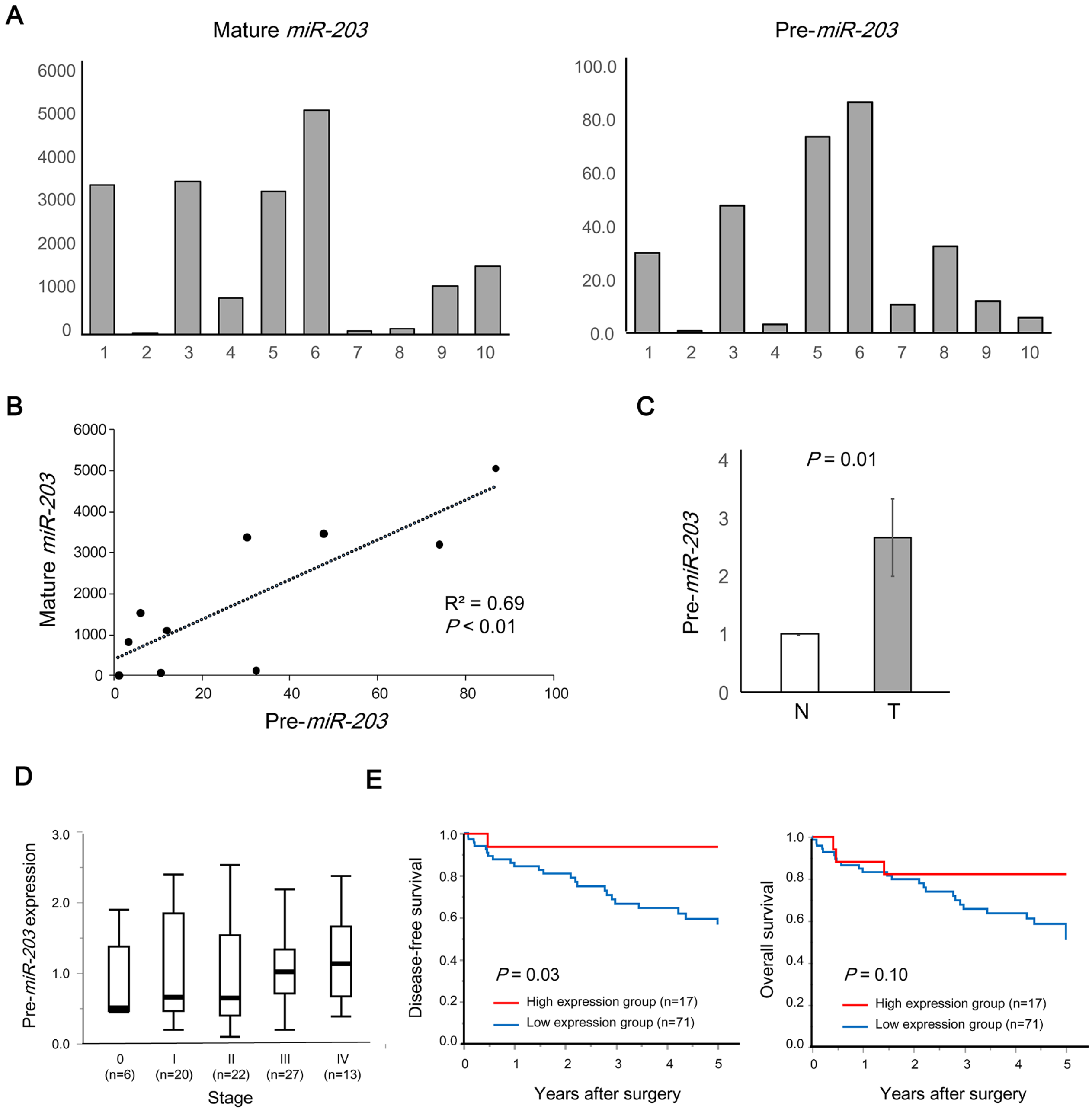

Supplementary Figure 3: Pre-*miR*-203 expression in various CRC cell lines and clinical significance of the expression in CRC tissues. **(A)** Quantification of mature or pre-*miR*-203 expression in various CRC cell lines by RT-qPCR. 1: Colo205; 2: Colo320DM; 3: DLD-1; 4: HCT116; 5: Lovo; 6: SW480; 7: SW620; 8: HT29; 9: RKO; 10: CaR-1. **(B)** Correlation between mature and pre-*miR*-203 expression in CRC cell lines. **(C)** Quantification of pre-*miR*-203 expression in CRC tissues by RT-qPCR. T: tumor tissue; N: normal colorectal tissue. **(D)** Pre-*miR*-203 expression in different TNM stages of CRC from Kyushu datasets. There was no statistical difference in pre-*miR*-203 expression among TNM stages. **(E)** Kaplan-Meier OS curves of 88CRC patients based on the pre-*miR*-203 expression in Kyushu datasets. Left: DFS; Right: OS.

Supplementary Table 1: Univariate and multivariate analysis of prognostic factors for DFS of CRC patients in Kyushu datasets (n=88)

Variables	Univariate analysis		Multivariate analysis	
	HR (95% CI[a])	*P*	HR (95% CI)	*P*
Histology (Not well/Well)	1.74 (0.77-4.31)	0.19		
Tumor size (≧5cm/<5cm)	2.32 (1.02-5.45)	0.04	0.96 (0.34-2.66)	0.44
Depth of invasion (≧SS[c]/≦MP[b])	20.02 (4.21-358.22)	<0.01	8.65 (1.26-180.00)	0.03
Venous invasion (+/-)	4.42 (1.85-9.98)	<0.01	3.92 (1.45-10.54)	<0.01
Lymphatic invasion (+/-)	2.70 (1.20-6.18)	0.02	0.94 (0.35-2.50)	0.90
Lymph node metastasis (+/-)	4.63 (1.99-11.98)	<0.01	1.20 (0.36-3.97)	0.76
Liver matastasis (+/-)	10.15 (4.18-23.28)	<0.01	4.51 (1.44-14.08)	0.01
Peritoneal dissemination (+/-)	39.11 (9.06-200.92)	<0.01	16.35 (2.93-108.60)	<0.01
Pre-*miR-203* in tumor tissue (high/low)	0.14 (0.01-0.68)	<0.01	0.17 (0.01-0.89)	0.03

[a]CI, confidence interval; [b]MP, muscularis propria; [c]SS, subserosa.

Supplementary Table 2: Relationship between clinicopathological factors and pre-*miR-203* expression in CRC tissues in Kyushu datasets (n=88) [a]**NA, not available;** [b]**MP, muscularis propria;** [c]**SS, subserosa.**

See Supplementary File 1

Supplementary Table 3: Patient characteristics

No	Age	Sex	Location	Size of primary tumor (cm)	T	N	H	P	M	Tissue-type	ly	v	Stage
1	48	F	RS[a]	2.8×2.0	3	0	0	0	0	Well	1	0	II
2	54	F	R[b]	3.0×2.8	2	0	0	0	0	Mod	1	0	I
3	70	M	S[c]	1.5×1.3	2	1	0	0	0	Well	1	0	IIIa
4	39	F	R-RS	9.0×8.5	4b	0	0	0	0	Por	1	0	II
5	62	M	R	7.6×4.8	3	0	0	0	0	Mod	1	1	II
6	39	F	S	4.5×4.0	3	0	0	0	0	Well	2	2	II
7	62	F	S<br>Liver	4.2×3.2	4a	3	1	0	1	Mod	1	1	IV
8	59	M	R<br>Liver	7.0×6.0	3	2	1	0	1	mod	1	2	IV
9	78	M	RS<br>Liver	6.0×2.5	3	2	1	0	1	mod	2	1	IV
10	69	F	S<br>Liver	4.8×3.1	3	1	2	0	1	mod	1	0	IV

[a]RS, rectosigmoid; [b]R, rectum; [c]S, sigmoid.
