# Supplementary material for: Circulating exosomal microRNA-203 is associated with metastasis possibly via inducing tumor-associated macrophages in colorectal cancer
Source: Oncotarget. 2017 Aug 7;8(45):78598–613. doi: 10.18632/oncotarget.20009 (PMC5667985; doi:10.18632/oncotarget.20009)
Supplement: Supplementary file 2 [file oncotarget-08-78598-s002.docx]

**Supplementary Table 2. Relationship between clinicopathological factors and pre-*miR-203* expression in CRC tissues in Kyushu datasets (n=88)**

| **Variables** | **Low (n=71)** | **High (n=17)** | ***P*** |
| --- | --- | --- | --- |
|  | **number (%)** | **number (%)** |  |
| Sex |  |  | 0.92 |
| Female | 26 (36.6) | 6 (35.3) |  |
| Male | 45 (63.4) | 11 (64.7) |  |
| Tumor size (cm) |  |  | 0.41 |
| ＜5 | 39 (54.9) | 11 (64.7) |  |
| ≧5 | 30 (42.3) | 5 (29.4) |  |
| NA^a^ | 2 (2.8) | 1 (5.9) |  |
| Histology |  |  | 0.98 |
| Well | 29 (40.8) | 7 (41.2) |  |
| Not Well | 42 (59.2) | 10 (58.8) |  |
| Depth of invasion |  |  | 0.05 |
| ≦MP^b^ | 23 (32.4) | 10 (58.8) |  |
| ≧SS^c^ | 48 (67.6) | 7 (41.2) |  |
| Venous invasion |  |  | 0.73 |
| (-) | 57 (80.3) | 15 (88.2) |  |
| (+) | 14 (19.7) | 2 (11.8) |  |
| Lymphatic invasion |  |  | 0.42 |
| (-) | 42 (59.2) | 12 (70.6) |  |
| (+) | 29 (40.8) | 5 (29.4) |  |
| Lymph node metastasis |  |  | 0.28 |
| (-) | 39 (54.9) | 12 (70.6) |  |
| (+) | 32 (45.1) | 5 (29.4) |  |
| Liver metastasis |  |  | 0.68 |
| (-) | 62 (87.3) | 16 (94.1) |  |
| (+) | 9 (12.7) | 1 (5.9) |  |
| Peritoneal dissemination |  |  | 0.33 |
| (-) | 67 (94.4) | 15 (88.2) |  |
| (+) | 4 (5.6) | 2 (11.8) |  |
| TNM Stage |  |  | 0.42 |
| I, II | 37 (52.1) | 11 (64.7) |  |
| III, IV | 34 (47.9) | 6 (35.3) |  |

^a^NA, not available; ^b^MP, muscularis propria; ^c^SS, subserosa.
